# Renal Transplantation with Simultaneous Aortoiliac Reconstruction Using a Polytetrafluoroethylene Vascular Graft for Severe Atherosclerosis

**DOI:** 10.1155/2018/8959086

**Published:** 2018-07-31

**Authors:** Go Anan, Koji Nanmoku, Masaki Shimbo, Masahiko Nagahama, Takaaki Kimura, Yasunaru Sakuma, Fumiyasu Endo, Yasuhiro Komatsu, Kazunori Hattori, Takashi Yagisawa

**Affiliations:** ^1^Department of Urology, St. Luke's International Hospital, Tokyo, Japan; ^2^Surgical Branch, Institute of Kidney Diseases, Jichi Medical University Hospital, Shimotsuke, Japan; ^3^Division of Nephrology, Department of Medicine, St. Luke's International Hospital, Tokyo, Japan

## Abstract

Studies on aortoiliac reconstruction for severe atherosclerosis with renal transplantation are limited. Here, we report a rare experience of the simultaneous reconstruction of the external iliac artery caused by severe atherosclerosis with polytetrafluoroethylene vascular graft and renal transplantation in a 55-year-old female; she was unable to undergo standard renal artery anastomosis to the right external iliac artery because of severe atherosclerosis, which would result in complete occlusion. Next, we directly anastomosed the donor renal artery to the polytetrafluoroethylene graft. After transplantation, delayed graft function occurred; therefore, the patient had to undergo hemodialysis. On day 7 after transplantation, her creatine level started to decrease. She was discharged from the hospital on the 14th day after transplantation. After 1 month, her serum creatinine level reduced to 1.12 mg/dL. After 3 years of transplantation, her serum creatinine level was 1.2 mg/dL. The simultaneous implantation of the polytetrafluoroethylene graft and renal transplantation was feasible as well as safe, with no infectious complications and stable renal function noted on long time follow-up. Although our case was rare, it emphasizes the need for transplant surgeons to gain surgical skills for vascular surgery using vascular grafts.

## 1. Introduction

Atherosclerosis of the iliac arteries occurs frequently in patients with end-stage renal disease who are awaiting renal transplants [1]. Several renal transplant candidates present with iliac artery atherosclerosis; this increases the technical difficulty of the procedure. As reported previously, 0.7%–3.6% of the recipients underwent aortoiliac reconstruction before, during, and after renal transplantation [2–8]. The indications for renal transplantation in these cases were controversial because of the difficulty associated with vascular dissection and the risk of infection. In addition, the vascular reconstruction timing remains controversial in both simultaneous and staged renal transplantation [2]. Here, we report a case of renal transplantation, wherein, owing to the emergency nature of the case, aortoiliac reconstruction was performed with the simultaneous implantation of a vascular graft.

## 2. Case Presentation

**Figure 1:**
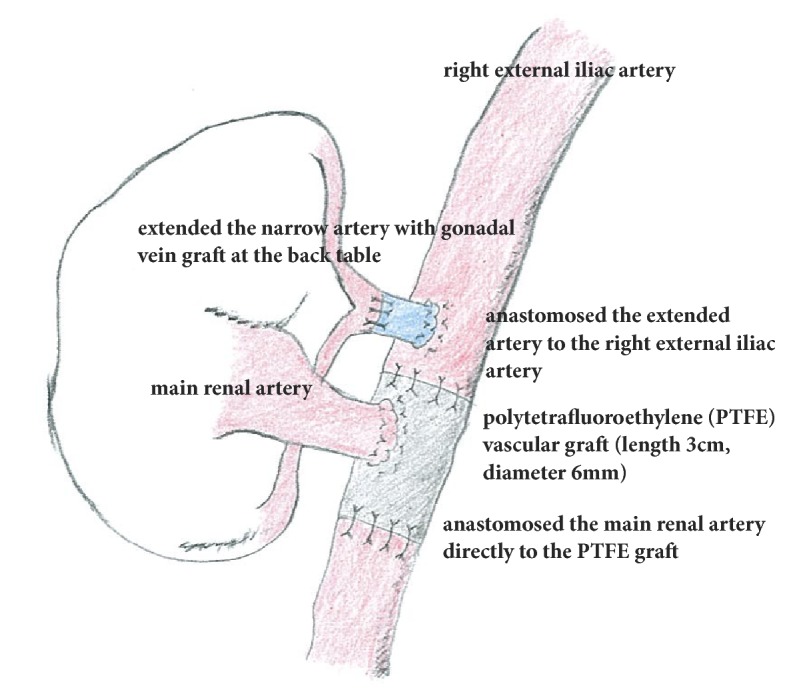
An illustration of aortoiliac reconstruction using polytetrafluoroethylene vascular graft.

The patient was a 55-year-old woman with end-stage renal disease of unknown cause who had been undergoing hemodialysis for the past 4 years before receiving a living-related renal transplant from her husband in September 2014. Before the transplantation, the recipient's computed tomography (CT) scan revealed moderate to severe atherosclerosis of the bilateral iliac arteries. Also, the donor's CT revealed two left renal arteries. For blood type-compatible and donor-specific antibody-positive recipients, desensitization was performed before transplantation with three sessions of double filtration plasmapheresis or plasma exchange and two doses of rituximab (100 mg). Immunosuppressive agents included triple immunosuppressive therapy with extended-release tacrolimus, mycophenolate mofetil, and methylprednisolone. Briefly, the transplant bed was made in the right iliac fossa. At the back table, there were two donor renal arteries, namely, the main artery and one narrow artery. Because the length of the narrow artery was short, it had to be extended using the donor's gonadal vein graft with end-to-end anastomosis using a 6-0 monofilament at the back table. Usually when there are two donor renal arteries, we connect the two arteries conjointly or side-to-end at the back table. However, as the length of one artery was short and the distance between the two arteries was large to connect, we thought that connecting the two arteries will enable anastomotic stenosis. Therefore, we decided to intracorporeally anastomose each renal artery to the iliac artery. The transplanted renal vein was anastomosed to the right external iliac vein side-to-end using a 5-0 monofilament with continuous sutures. First, considering the position of the two arteries, we decided to anastomose the extended artery to the right external iliac artery side-to-end using a 6-0 monofilament with continuous sutures. Second, before anastomosing the main artery to the iliac artery, we punched out the external iliac artery using an aorta punch; however, the cavity in the artery was narrow. Firstly, we performed endarterectomy to create sufficient space for anastomosis with the renal artery. However, we could not make a sufficient cavity to ensure adequate blood flow. Therefore, we decided to reconstruct the external artery using a polytetrafluoroethylene (PTFE) vascular graft to achieve adequate blood flow. We were unable to undertake the standard renal artery anastomosis to the right external iliac artery owing to severe atherosclerosis, which would result in complete occlusion. After reconstructing the external artery with the PTFE vascular graft (length, 3 cm; diameter, 6 mm), the transplanted main renal artery was anastomosed directly to the PTFE graft side-to-end using a 5-0 monofilament with continuous sutures (Figure 1). The surgical procedure lasted for 9 h and 14 min; the total ischemic time was 3 h and 20 min, and we could not confirm the first urine during the operation. The total bleeding volume was 450 mL; the same volume was transfused during the transplantation.

**Figure 2:**
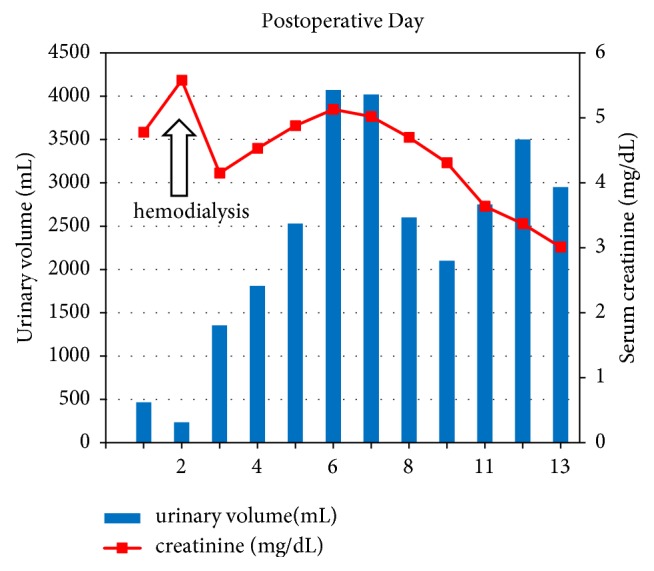
The clinical course of urinary volume and renal function.

After transplantation, delayed graft function occurred, for which the patient had to undergo hemodialysis session on the third day after transplantation. Seven days after the transplantation, her serum creatinine level started to decrease (Figure 2). The patient was discharged from the hospital on the 14th day after transplantation. Her serum creatinine level had reduced to 3.01 mg/dL on the day of discharge. Furthermore, after 1 month of discharge, her serum creatinine level reduced even further to 1.12 mg/dL. Presently, after 3 years of transplantation, her serum creatinine level is stable (1.2 mg/dL). She showed no sign of arterial graft infection after the renal transplantation. As she received an internalized antiplatelet agent before the transplantation because of coronary disease, internal resumption was resumed from seven days after transplantation to date.

## 3. Discussion

The number of renal transplant candidates with extended indications, such as older age and advanced atherosclerosis, is currently increasing [1, 2]. This has increased the number of cases that are difficult to treat using vascular anastomosis. However, studies on aortoiliac reconstruction with renal transplantation are limited. In transplant recipients with iliac artery atherosclerosis, it is possible to perform additional vascular procedures simultaneously, before performing transplantation in selected cases [2–8]. In the simultaneous aortoiliac reconstruction group, perioperative surgical complications, such as bleeding and infection, have been noted. No perioperative mortality was related to the additional vascular procedure [2]. Moreover, simultaneous aortoiliac reconstruction was not associated with increased risk of delayed graft function or with a longer hospital stay [2]. However, in our case, delayed graft function occurred, necessitating the need for hemodialysis. Contrarily, another study on 11 patients reported simultaneous aortoiliac reconstruction, wherein two grafts were lost because of bleeding from the anastomotic site and primary nonfunction resulting from prolonged warm ischemia [3]. No infectious complications of the vascular graft were noticed during the 4 years in all 11 renal recipients [3]. Contrarily, one recipient died because of prosthetic graft infection secondary to a urinary tract fistula [4].

Considering the advantages and disadvantages, we have summarized the differences in the vascular procedures before or during transplantation [2–8]. Several advantages were noted in simultaneous transplantation with vascular reconstruction. First, no technical difficulties in the dissection caused by reoperation were noticed. Second, the hospitalization time and cost are reduced as compared with those of separate operations. Third, general anesthesia is required only once; thus, risks caused by anesthesia are decreased. There were also several disadvantages. First, the operation procedure was complex and difficult to perform. Second, there was an inherent risk of prosthesis infection in immunosuppressed patients. Third, there was a risk of graft function because of the long ischemic time and extra procedure.

At our institution, up to two renal arteries are usually selected in the left kidney as donors to secure a long renal vein. In the case of two renal arteries, we normally connect the two arteries conjointly or side-to-end at the back table. Therefore, we selected the left kidney as the donor in this case. It is notable that the increased use of kidneys with multiple renal arteries does not affect any clinical outcome of the recipient besides total ischemia time [9].

In our case, the patient showed no symptoms of vascular disorders. At our institution, we do not usually perform enhanced CT scan and vascular 3D reconstruction before transplantation. Thus, we could not predict severe arteriosclerosis of the external iliac artery from nonenhanced CT scan in this case. In mild arteriosclerosis cases, we normally perform endarterectomy to make sufficient space for successful anastomosis with the renal artery. If severe arteriosclerosis of the iliac artery is predicted before transplantation, we consider anastomosis to the iliac artery on the contralateral side and the common iliac artery. During the transplantation, we could not make sufficient space to anastomose the renal artery after endarterectomy; therefore, we finally decided to reconstruct the external artery using the PTFE vascular graft. We successfully performed the simultaneous implantation of PTFE graft with stable renal function and no infectious complications. From our experience, we noted some important points for successful aortoiliac reconstruction performed simultaneously with renal transplantation. First, surgeons should always be prepared for sudden vascular reconstruction. Renal transplant surgeons should continuously gain and enhance their skills in performing anastomosing prosthesis. Second, to avoid prosthesis infection, it is important to ensure hemostasis and prevent urinary and lymph fistulas.

## 4. Conclusion

The simultaneous implantation of the PTFE graft with renal transplantation because of severe atherosclerosis is feasible and safe, because stable renal function and no infectious complications were noted in our patient.
